# Supplementary material for: H3K9 Promotes Under-Replication of Pericentromeric Heterochromatin in Drosophila Salivary Gland Polytene Chromosomes
Source: Genes (Basel). 2019 Jan 29;10(2):93. doi: 10.3390/genes10020093 (PMC6409945; doi:10.3390/genes10020093)
Supplement: Supplementary file 1 [file genes-10-00093-s001.pdf]

Supplementary Materials

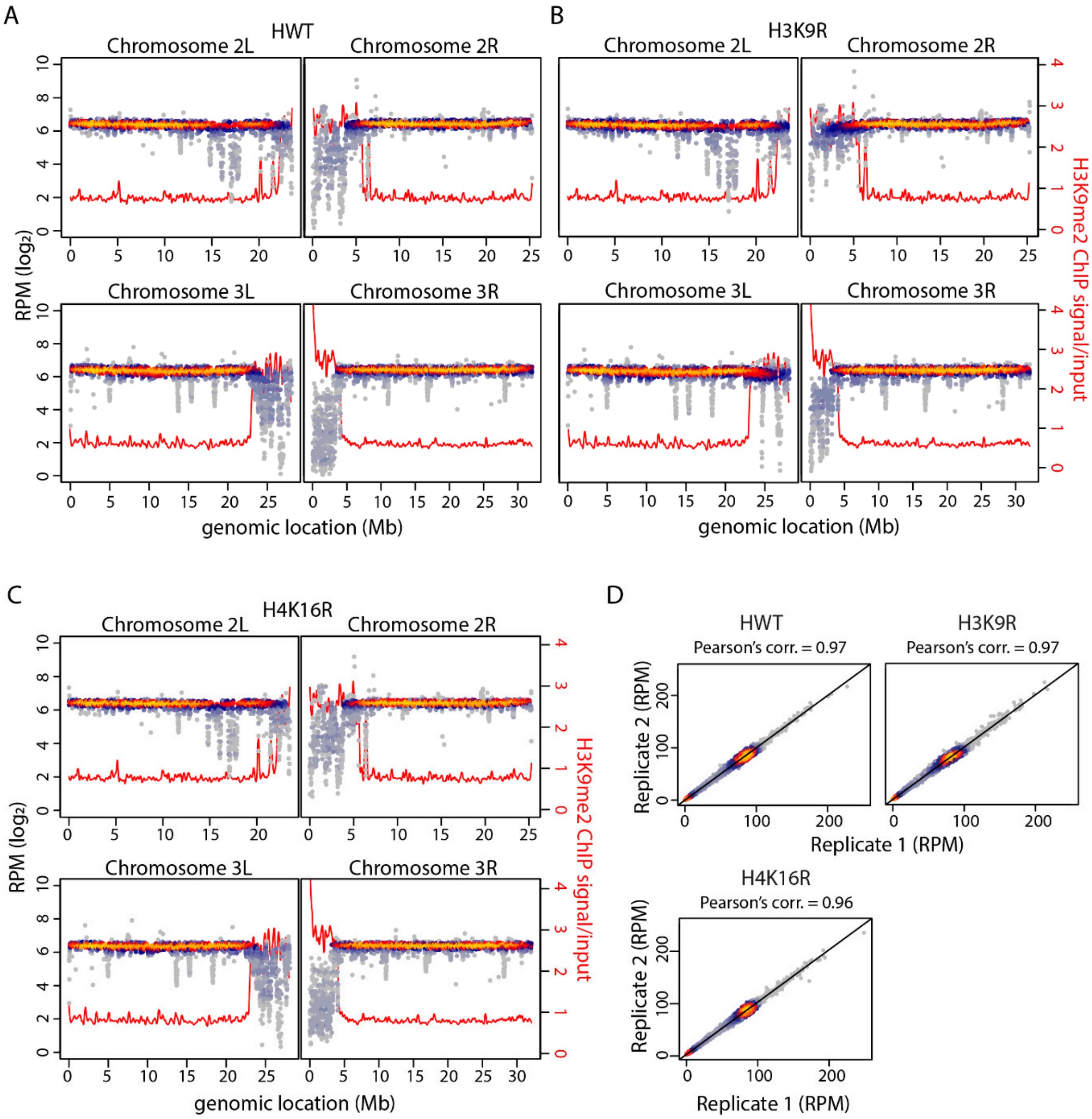

Figure S1. DNA copy number in pericentric heterochromatin is elevated in *H3K9R* mutants. **A**–**C**) Heatscatter plot of A) *HWT* normalized copy number ($\log_2$), **B**) *H3K9R* normalized copy number ($\log_2$) and **C**) *H4K16R* normalized copy number ($\log_2$) at 10kb windows along Chromosomes 2 and 3. LOESS regression line of modENCODE H3K9me2 ChIP signal is shown in red (GSE47260). **D**) Heatscatter plot comparing normalized signal at 10kb windows of two replicates each for *HWT*, *H3K9R* and *H4K16R* genotypes along all major chromosome scaffolds (Chromosomes 2L, 2R, 3L, 3R, 4 and X). Pearson's correlation value is indicated.
